# Failed Suicide Attempt: From the Forehead to the Mediastinum

**DOI:** 10.1002/ccr3.71150

**Published:** 2025-10-14

**Authors:** N. Jacques, L. Boisliveau

**Affiliations:** ^1^ Intensive Care Unit University Hospital Saint Pierre La Réunion France; ^2^ Radiology Department University Hospital Saint Pierre La Réunion France

**Keywords:** failed suicide attempt, mediastinitis, neurocognitive disorders, penetrating cranial injuries, retropharyngeal space, suicide behavior

## Abstract

Outside of gunshot wounds, suicidal behavior involving the head is rare. This case presents a patient with a favorable outcome despite a risk of mediastinitis. The anatomy of the deep cervical spaces and the absence of an anatomical barrier to the mediastinum are important to know to prevent this complication.

An 81‐year‐old man with no prior medical history was hospitalized for recent neurocognitive disorders associated with depressive syndrome, anorexia, and irritability. During his hospitalization, he attempted suicide by directly impacting his forehead against a vertically positioned pencil placed on the table (Figure 1). The CT scan showed the pencil penetrating from the right frontal bone through the frontal sinus (where the pencil lead had come loose) and the ethmoidal cells (Figure 2), then entering the oropharynx and crossing the posterior pharyngeal wall (Figure 3) without vascular or neurological lesions but with the presence of air in the retropharyngeal space and upper mediastinum. The patient was managed by retrograde removal of the pencil under general anesthesia (Figure 4). Post‐operative care consisted of prophylactic Amoxicillin/Clavulanate, placement of a nasogastric tube to protect the pharyngeal suture, and resumption of oral intake on Day 5. There is no anatomical barrier between the mediastinum and the neck [1]. The major risk is the spread of a cervical infection along the deep cervical fascia to the mediastinum. In cases of strong clinical suspicion, mediastinitis secondary to infection of the cervical soft tissues should be ruled out. In our patient's case, the retropharyngeal spaces were involved, leading to the use of Amoxicillin/Clavulanate as prophylactic antibiotic therapy to prevent mediastinitis [2]. The recovery was good, without evidence of mediastinitis. Suicidal behavior involving the head is rare, especially outside of gunshot wounds. Only a few similar cases have been reported in the literature. Moreover, penetrating cranial injuries typically result in death due to the density of critical vascular and neurological structures in this anatomical region. The survival of the patient in this case, along with a favorable outcome, makes this report especially noteworthy. Finally, the sudden onset, the severity of the act, and the recent neurocognitive disorders prompted an extensive search for an organic etiology, such as anti‐NMDA receptor encephalitis, depending on the clinical context [3].

**FIGURE 1:**
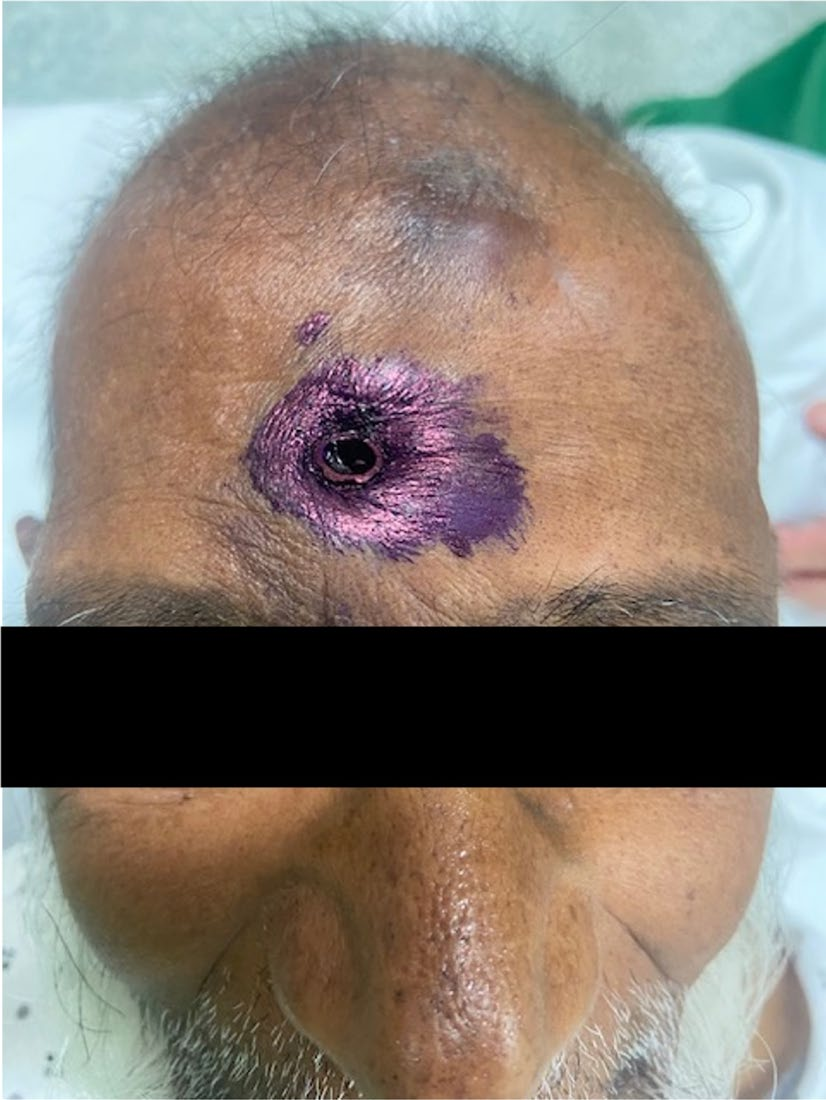
Entry point of the foreign material.

**FIGURE 2:**
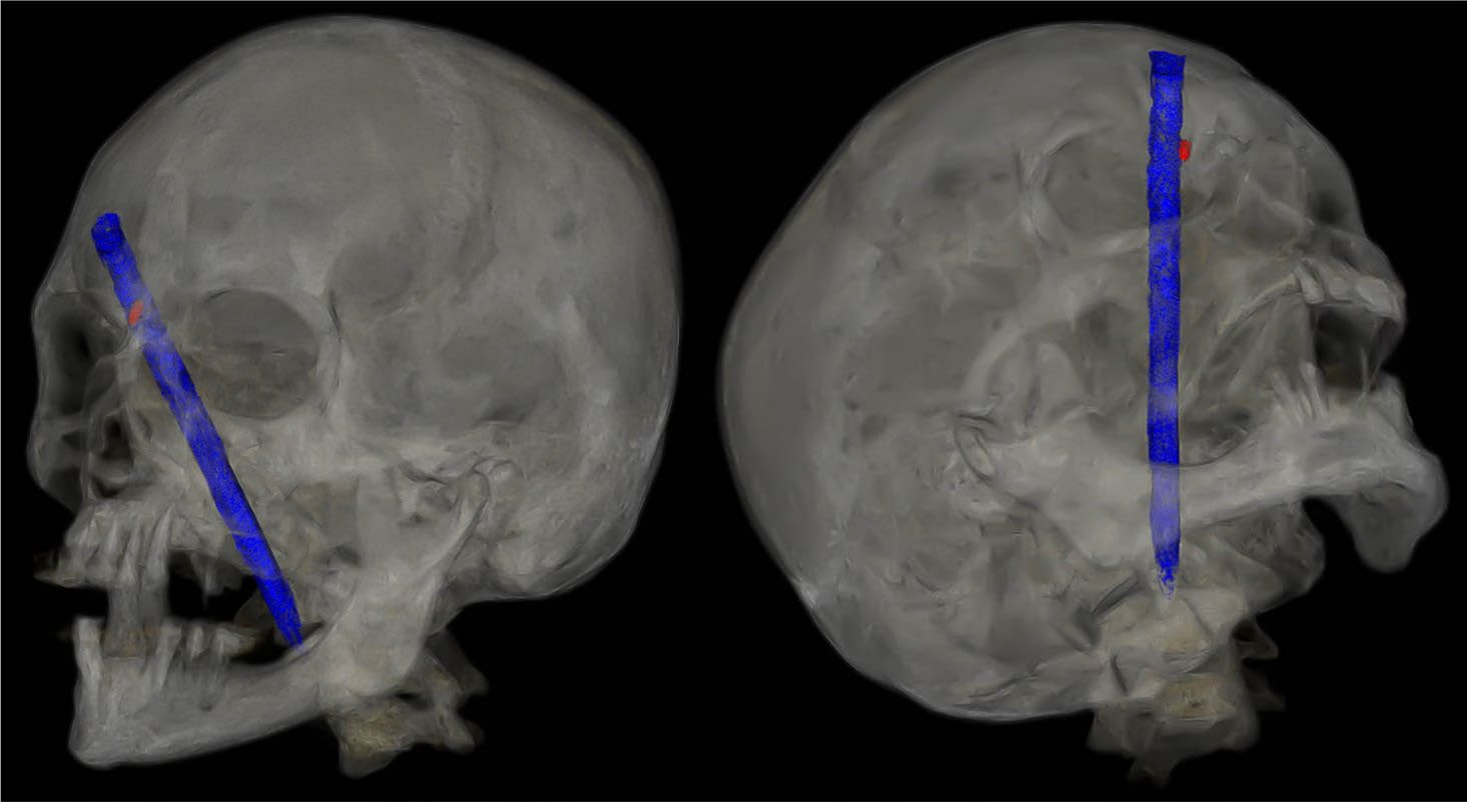
3D CT scan images showed in blue color the trajectory of the pencil from the forehead to the upper mediastinum and in red color the position of the pencil lead.

**FIGURE 3:**
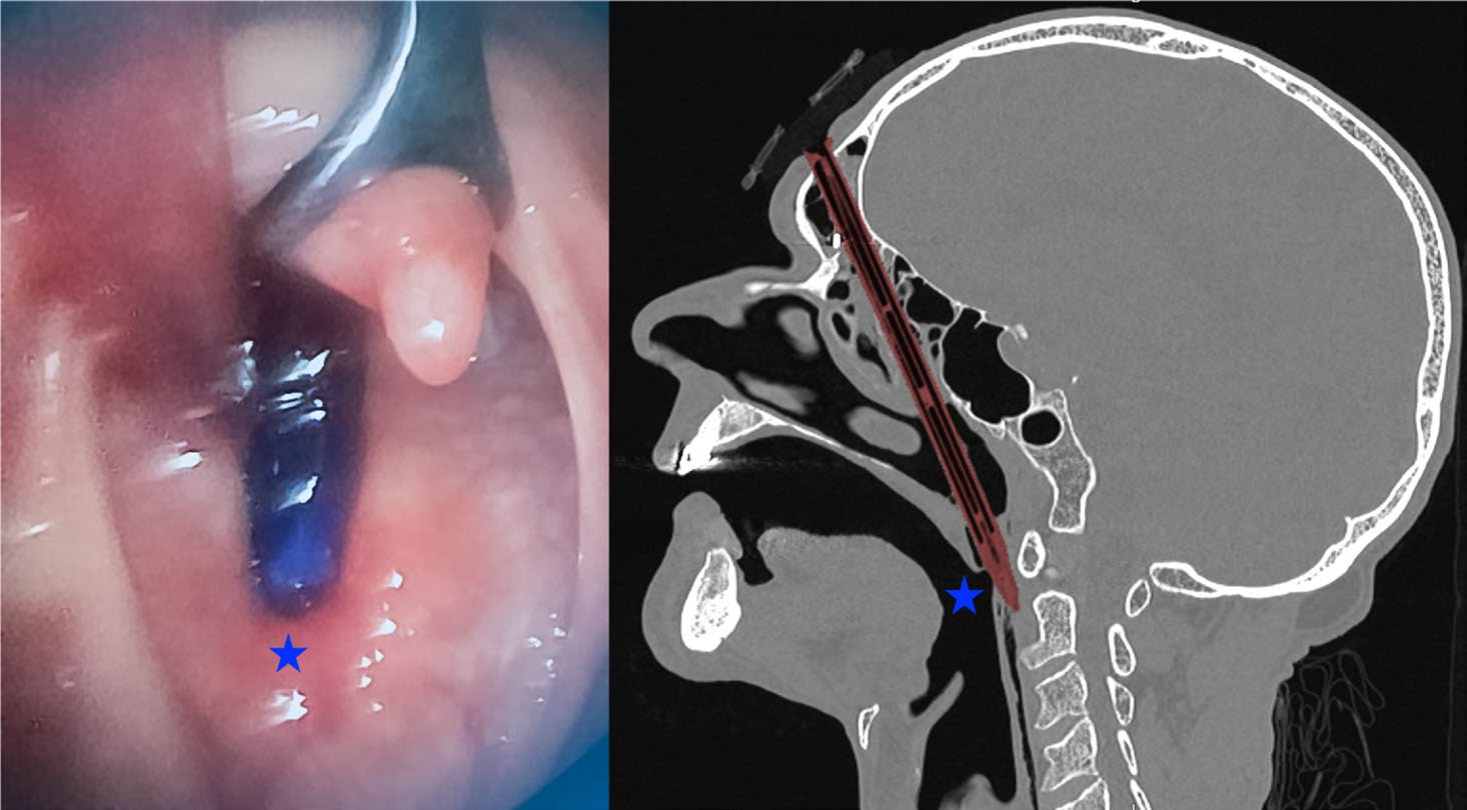
Pencil entry point in the posterior pharyngeal wall (blue stars).

**FIGURE 4:**
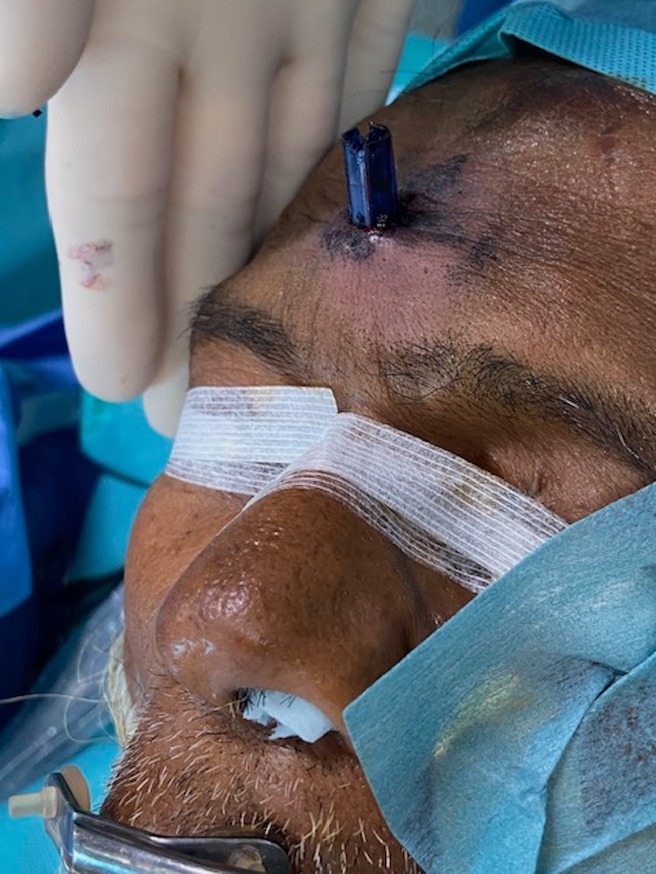
Retrograde removal of the pencil under general anesthesia.

## Author Contributions

**N. Jacques:** conceptualization, writing – original draft, writing – review and editing. **L. Boisliveau:** resources, validation.

## Consent

Written informed consent was obtained from the patient.

## Conflicts of Interest

The authors declare no conflicts of interest.

## Data Availability

Anonymized data available on request from the authors.
